# Lateralization influences contest behaviour in domestic pigs

**DOI:** 10.1038/s41598-018-30634-z

**Published:** 2018-08-14

**Authors:** Irene Camerlink, Sophie Menneson, Simon P. Turner, Marianne Farish, Gareth Arnott

**Affiliations:** 1 Animal Behaviour & Welfare, Animal and Veterinary Sciences Research Group, Scotland’s Rural College (SRUC), West Mains Rd., Edinburgh, EH9 3JG UK; 2 Institute for Global Food Security, School of Biological Sciences, Queen’s University, Belfast, BT9 7BL UK; 3 Present Address: Institute for Animal Husbandry and Animal Welfare, University of Veterinary Medicine Vienna, Vienna, Veterinarplatz 1, 1210 Austria

## Abstract

Cerebral lateralization, i.e. hemispheric asymmetries in structure and function, relates in many species to a preference to attack from their left. Lateralization increases cognitive capacity, enabling the simultaneous processing of multiple sources of information. Therefore, lateralization may constitute a component of fighting ability (Resource Holding Potential), and/or influence the efficiency of information-gathering during a contest. We hypothesized that lateralization will affect contest outcome and duration, with an advantage for more strongly lateralized individuals. In 52 dyadic contests between weight-matched pigs (*Sus scrofa;* n = 104; 10 wk age), the direction of orientation towards the opponent was scan sampled every 10 s. Laterality indexes (LI) were calculated for the direction and strength of lateralization. Up to 12.5% of the individuals showed significant lateralization towards either the right or left but lateralization was absent at the population level. In line with our hypothesis, animals showing strong lateralization (irrespective of direction) had a shorter contest duration than animals showing weak lateralization. Winners did not differ from losers in their strength or direction of lateralization. Overall the results suggest that cerebral lateralization may aid in conflict resolution, but does not directly contribute to fighting ability, and will be of value in the study of animal contests.

## Introduction

Cerebral lateralization is widespread in the animal kingdom. While formerly it was thought that only humans showed brain lateralization^1,2^, with the right and left hemisphere having their own functions, it has since been demonstrated widely across both vertebrates^3–5^ and invertebrates^6–8^. The left hemisphere is related to routine tasks, the parasympathetic nervous system, visual stimuli and responding to prey or to food, amongst other roles. The right hemisphere has been associated with the response of the sympathetic nervous system, emergency situations, novel stimuli, fear, aggression, withdrawal, empathy, social recognition and responses to conspecifics^5^. In vertebrates, the hemispheres operate contralaterally^9,10^, meaning that the majority of the functions of the left hemisphere are expressed through the right side of the body and the functions of the right hemisphere are expressed through the left side of the body. This contralateral organisation of the forebrain is not present in invertebrates, where the brain-body connections are predominantly ipsilateral (i.e. on the same side of the body) although the central nervous system of invertebrates does have bilateral connections showing parallels with vertebrates^10,11^. In both vertebrates and invertebrates, the use of the left/right limb or eye has been used in research as an indicator of cerebral lateralization.

Lateralization can be studied for its direction, with either an individual-level or population-level preference to use left or right in a certain situation, and for the strength of lateralization, indicating the degree of integration between the hemispheres^12^. Lateralization at the individual level refers to the lateralization that an individual shows whereas lateralization at the population level refers to an overall tendency of the population to move in a certain direction or to perform tasks with a preference for either their left or right side^12^, such as handedness in humans^2,13^.

While numerous studies have documented lateral biases in escalated attack behaviour (reviewed in^5^), detailed examination of influences of lateralization on whole contests including contest duration and fight success has remained largely unexplored, in particularly in large mammals. Austin and Rogers^14^ showed that feral stallions (*Equus caballus*) show left eye bias during fights, but did not record the effect of lateralization on fight outcome. In studies on fallow deer (*Dama dama*) it has been suggested that lateralization contributes to contest resolution, but evidence is inconclusive and shows a right-sided bias during contests^15–18^. Research on lateralization during contests in invertebrates is growing (fruit fly *Bactrocera oleae*^19^; fruit fly *Ceratitis capitata*^20^; blowfly *Calliphora vomitoria*^21^).

Contest theory models make predictions that reflect decisions made in aggressive encounters^22,23^. If individual lateralization contributes to fighting strategies and/or to an animal’s fighting ability, termed resource-holding potential (RHP)^24^, then the study of animal contest behaviour can benefit from taking into account the effects of lateralization. Across vertebrates non-damaging agonistic display is mostly performed with the right eye facing the opponent (information being processed in the left hemisphere) and attacks are preferentially made with the left eye facing the opponent (horses^14^; lizards^25^; frogs^26^). One of the suggested advantages of stronger lateralization, comprising a higher degree of hemispheric specialisation, is that different types of information can be processed simultaneously^12^. Hence, it is suggested that more strongly lateralized individuals may have cognitive advantages under certain circumstances (e.g. with performing different tasks simultaneously). For example, more strongly lateralized chicks (*Gallus gallus domesticus*) were better able to simultaneously find food while remaining vigilant for a predator^27^. During animal contests, multiple signals may be sent and received by the opponents (i.e. multimodal signalling^28^), which may activate cognitive processes related to various brain regions (e.g. for olfaction, vision, and auditory cues). It may therefore be that animals which are strongly lateralized are better at processing information about their opponent and thus would be better at assessing their opponent’s fighting ability. Contestants may utilize the advantage to process information rapidly either to win the contest or to retreat sooner^29^, which suggests that lateralization could contribute to an animal’s fighting ability. Consequently, the outcome of dyadic contests might be reached sooner and physical costs of aggression minimized.

Here we studied lateralization in dyadic contests between pigs, building on our research that has used a game theoretical approach^30^, examining if the strength of lateralization affects contest duration and contest outcome; the main measures required in game theory models^23,31,32^. Agonistic behaviour between pigs includes displays that require the animal to assume a right or left sided position (e.g. parallel walking) and may therefore have developed to favour one direction. Moreover, aggression between pigs is a considerable welfare issue^33^ whereas knowledge of lateralization has repeatedly been proposed as a tool for improving animal welfare^34,35^. We aimed to test whether the strength of lateralization contributes to the contest duration and contest outcome. We hypothesized that more strongly lateralized animals would be better at processing information about the opponent and with this information would be more likely to win a contest, whereas more strongly lateralized losers would retreat sooner; both situations resulting in a shorter contest duration.

## Results

**Figure 1:**
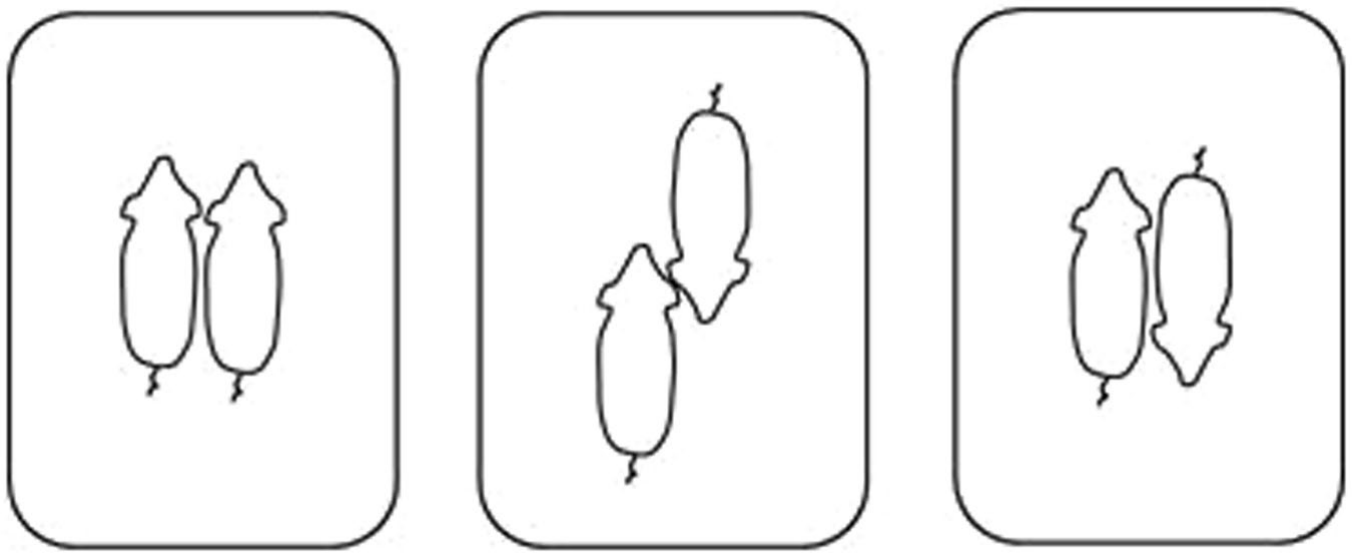
Opponent configurations. Positions recorded during the contest. Parallel, head-to-head and head-to-tail, respectively. Illustration by S. Menneson.

Dyadic contests were analysed for the frequency of left and right orientation towards the opponent (Fig. 1), from which a laterality index (LI) was calculated, providing information on the direction of lateralization (range −1 to +1), and the absolute laterality index (ABLI), which is a standardized index to represent the strength of lateralization (range 0 to +1). LI and ABLI were calculated over the whole contest as well as during display behaviour, non-damaging aggression and damaging aggression separately (behaviours described in Table 1). Significance of individual preferences for left or right was determined using z-scores.

**Table 1:** Ethogram used for obtaining the duration of behaviours.

Behaviour	Description
Display	Ritualized agonistic behaviour, largely non-contact. Includes mutual investigation, parallel walking, shoulder pressing, and heads up position
Non-damaging aggression	Agonistic behaviour where opponents make forceful contact without causing significant injury. Includes here only the mutual behaviours pushing and nose wrestling
Damaging aggression	Opponents retaliate to each other’s aggressive act (biting and pushing during a fight) within 5 s
Other behaviour	All behaviour other than above

All behaviours (except ‘other’) were mutual, meaning that the duration was the same for the winner and loser.

**Figure 2:**
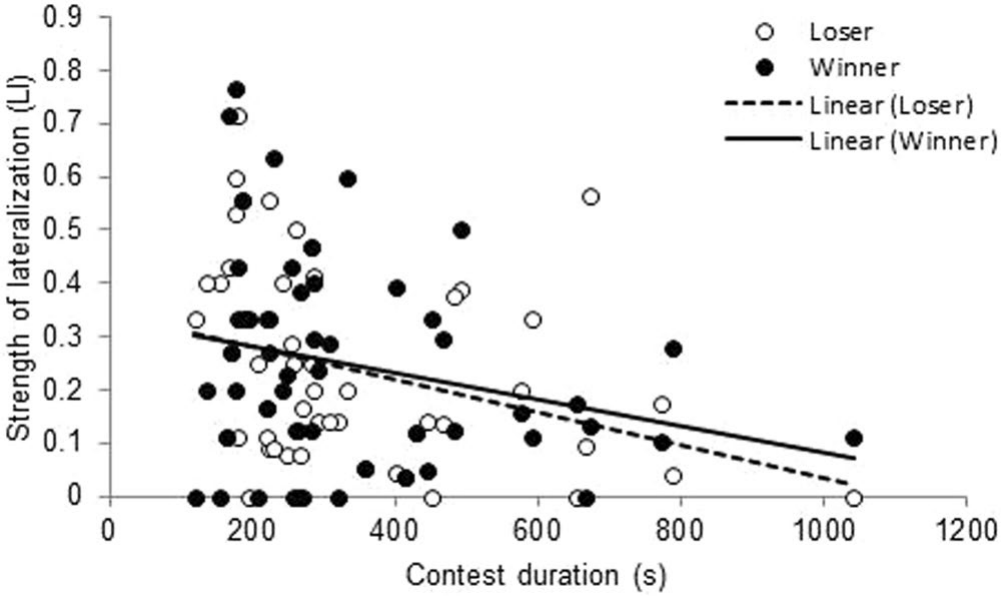
Strength of lateralization. Relationship between strength of lateralization (LI) and contest duration for winners (black dots and solid trend line) and losers (open circles and dotted trend line).

Contests lasted on average 412 ± 29 s (~7 min; range 119–1800 s), with 78 ± 5 s spent on display behaviour (0–235s), 46 ± 7 s on non-damaging aggression (0–313s), and 67 ± 5 s on damaging aggression (0–204s). The total contest duration was shorter when the loser was more strongly lateralized (*b* = −0.8 ± 0.4 log s; *F*_1,24_ = 4.82; *P* = 0.04; Fig. 2) and tended to be shorter when the winner was more strongly lateralized (*b* = −0.6 ± 0.3 log s; *F*_1,24_ = 3.64; P = 0.07; Fig. 2). This is in line with the hypothesis that contest duration may be shorter when the loser is more strongly lateralized.

Winners did not differ from losers in their direction of lateralization (LI) or strength of lateralization (ABLI) (Table 2). This was particularly true for behaviour where the position of both opponents is related to each other, such as during fights where animals may interlock. This suggests that the strength and direction of lateralization does not contribute to the ability of pigs to be successful in fighting, and therefore does not contribute to their RHP.

**Table 2:** Means with SE for the direction (LI) and strength (ABLI) of lateralization for winners and losers for the overall contest, during display behaviour and during damaging aggression.

Measure of lateralization	Winners (*n* = 52)	Losers (*n* = 52)	*t-*value	*P*-value
LI (direction: −1 to + 1)				
over total contest	0.02 ± 0.04	−0.05 ± 0.04	−1.02	0.31
during display	−0.09 ± 0.07	−0.04 ± 0.07	0.57	0.58
during aggression	0.00 ± 0.09	0.02 ± 0.08	0.10	0.92
ABLI (strength: 0–1)				
over total contest	0.25 ± 0.03	0.24 ± 0.03	−0.24	0.81
during display	0.32 ± 0.04	0.36 ± 0.04	0.55	0.59
during aggression	0.41 ± 0.05	0.41 ± 0.04	0.09	0.93

Strongly lateralized winners tended to spend a smaller percentage of time on display behaviour (*b* = −14.6% per 0.1 ABLI; *F*_1,20_ = 3.04; *P* = 0.09) but this was unaffected by the overall ABLI of the loser (*F*_1,20_ = 0.19; *P* = 0.67). The overall strength of lateralization did not affect the percentage of time spent on non-damaging aggression (winner *F*_1,22_ = 0.06; *P* = 0.81; loser *F*_1,22_ = 0.59; *P* = 0.45) or fighting (winner *F*_1,20_ = 0.13; *P* = 0.73; loser *F*_1,20_ = 1.73; *P* = 0.20). The strength of lateralization shown specifically during display behaviour did not affect the percentage of time spent on display behaviour (winners *F*_1,20_ = 0.84; *P* = 0.37; losers *F*_1,20_ = 1.40; *P* = 0.25). Similarly, the strength of lateralization recorded during fighting did not affect the percentage of time spent on this behaviour (winners *F*_1,20_ = 1.64; *P* = 0.21; losers *F*_1,20_ = 2.07; *P* = 0.17). Not all contests included fighting behaviour, and out of the 52 contests 14 did not include a fight. The occurrence of a fight was unrelated to the strength of lateralization of the winner (*F*_1,20_ = 0.15; *P* = 0.70) and the loser (*F*_1,20_ = 2.95; *P* = 0.101).

**Figure 3:**
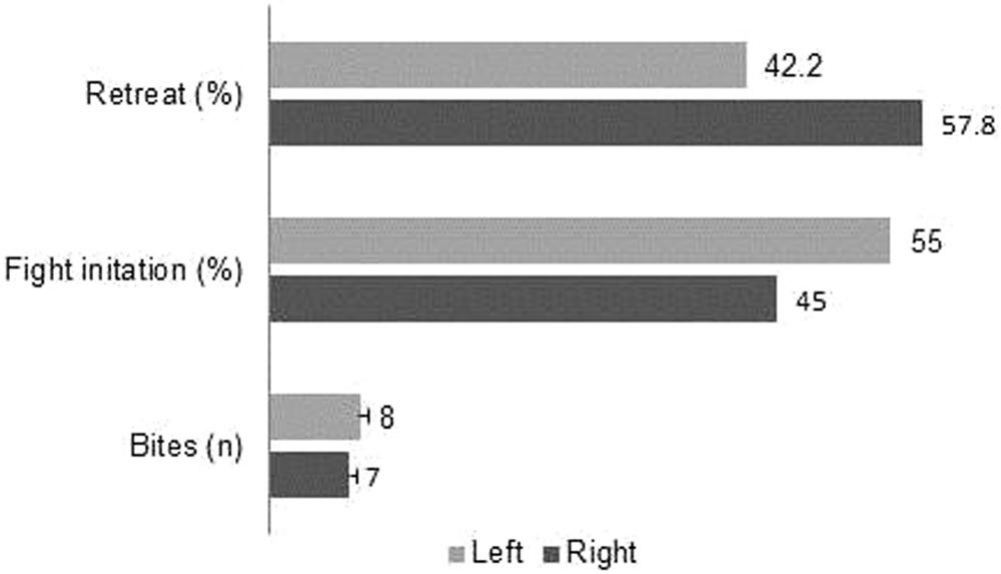
Left/right orientation during contest behaviour. Number of bites given while oriented with the head left or right towards the opponent, and the percentage of left/right orientations when initiating a fight (side facing the opponent) and when retreating from the winning opponent (side facing the winner). The number of bites is the average number of bites (mean ± SE) per dyad.

The direction towards the opponent when biting, initiating a fight and during retreat was recorded for winners and losers. There was no difference in the frequency of left and right for the number of unilateral bites (outside mutual fighting; range 0–66 bites/contest; *t* = −1.14, df = 103, *P* = 0.26) or the orientation at the onset of a fight (recorded 92 times; *t* = −1.04, df = 103, *P* = 0.30). The direction in which the loser retreated from the winner occurred with equal frequency from the right and left (*t* = 1.55, df = 100, *P* = 0.12; Fig. 3).

The LI of all observations during the contests combined showed a normal distribution (Shapiro-Wilk (SW) 0.98; Skewness (S) 0.39; Kurtosis (K) −0.21), with an average of −0.01 ± 0.03 and a range of −0.60 to 0.76. The average did not differ from zero (*t* = −0.45; df = 103; *P* = 0.66), indicating no population level lateralization. The LI calculated for specific behaviours showed similar distributions; the LI of display behaviour (mean −0.07 ± 0.05; SW 0.97; S 0.10; K 0.15) and damaging aggression (mean 0.01 ± 0.06; SW 0.97; S −0.04; K −0.40), did not differ from 0 (display *t* = −1.38; df = 57; *P* = 0.17; damaging aggression *t* = −0.14; df = 59; *P* = 0.89).

The average strength of lateralization, or absolute LI (ABLI), was 0.25 ± 0.02 (range 0–0.76) for the total contest, 0.34 ± 0.03 during display, and 0.41 ± 0.03 during damaging aggression. Individual z-scores revealed that, over all observations, 8.5% of the animals showed significant preference for left (5.4%) or right (3.1%) (z-score > 1.96 or < −1.96; *P* < 0.05). For display behaviour only 3.2% of the animals showed a significant preference but for aggressive behaviour this was 12.5% (significant right preference 5.4%; left preference 7.1%).

**Figure 4:**
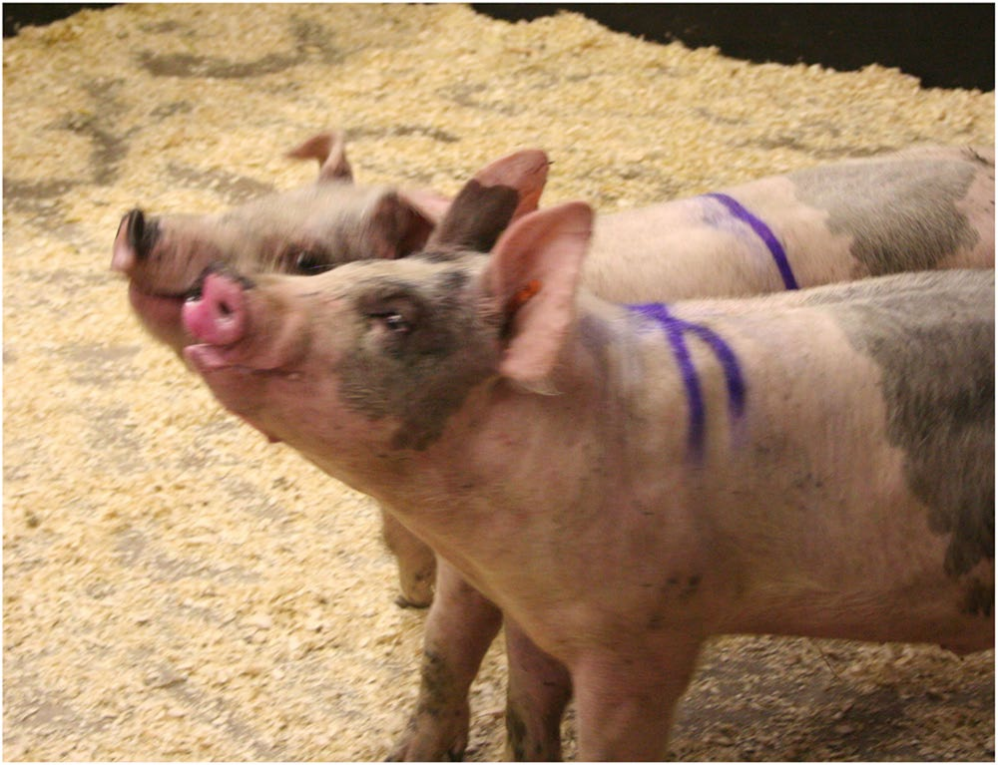
Lateralization in behaviour. Parallel walking between opponents, scored as parallel position. Photo by M. Farish.

The parallel position (Fig. 1) was mostly observed when opponents were engaged in parallel walking, which is a ritualized form of display behaviour where opponents move side-by-side (depicted in Fig. 4). The direction of lateralization (LI) during the parallel position showed a normal distribution (SW 0.98; S 0.02; K −0.09) with an ABLI of on average 0.35 ± 0.03. During the parallel position 5% of the population had a left bias and 5% had a right bias (z-score > 1.96 or < −1.96; *P* < 0.05). The ABLI was unrelated to the contest duration (*F*_1,25_ = 1.30; *P* = 0.27) and the time spent in display behaviour (*F*_1,23_ = 0.03; *P* = 0.86).

The LI was unrelated to sex (*F*_1,82_ = 0.10; *P* = 0.75) and body weight (*F*_1,82_ = 0.07; *P* = 0.79), but for display behaviour specifically males were more oriented with their left towards the opponent (−0.16 ± 0.08), whereas females were more oriented with their right (0.05 ± 0.09) (*F*_1,39_ = 5.33; *P* = 0.03). The ABLI overall was unrelated to sex (*F*_1,83_ = 0.93; *P* = 0.34) and weight (*F*_1,83_ = 0.95; *P* = 0.33), but during display behaviour females tended to be more strongly lateralized than males (female 0.38 ± 0.06; males 0.28 ± 0.06; *F*_1,83_ = 2.82; *P* = 0.09).

## Discussion

Lateralization has been widely studied across species, including during agonistic behaviour. The role of lateralization in the outcome of animal contests, i.e. fight duration and fight success, has however remained largely unexplored. Here we studied the influence of lateralization on contest dynamics in pigs, testing predictions derived from contest theory^32^, evaluated by examining contest duration and outcome. Lateralization of behaviour, analysed from 52 dyadic contests, revealed that contests were shorter when animals showed stronger lateralization. However, the direction and strength of lateralization did not differ between winners and losers, meaning that it does not contribute directly to fighting ability. Up to 12.5% of the pigs showed significant side bias in their agonistic behaviour at the individual level, but lateralization was absent at a population-level.

We found that strongly lateralized animals had a shorter contest duration. This suggests that these animals were more efficient in their information processing and could thus sooner resolve a conflict. Although the percentage of significantly lateralized individuals was small, the percentage corresponds to other studies in which no population level lateralization was found^36^. Furthermore, strength of lateralization is on a scale rather than a binary trait, and the significant linear relationship with contest duration (Fig. 2) is therefore revealing, that those individuals lateralized to a lower (non-significant) degree can still benefit. Overall, the data shows that strongly lateralized animals do benefit from a shorter contest duration but that a certain degree of unpredictability in the agonistic responses is still favoured even within individuals^37^. Thus, there may be a trade-off with costs and benefits of lateralization in contest scenarios^16^. This could include speed-accuracy trade-offs in decision making, with more strongly lateralized individuals making more rapid but not necessarily accurate decisions^38^. Consistent with this is the fact that lateralization did not contribute to RHP, in terms of fight outcome, in the current study.

During their agonistic interactions animals give signals involving multiple sensory modalities (i.e. multimodal signalling^28^). For pigs these include visual signals such as piloerection^39^, vocal cues^40^ and scent cues to enable individual recognition^41^. Stronger lateralization could potentially have facilitated better processing of such information coming from the opponent, resulting in a quicker decision to fight or retreat. In the only other examination of lateralization and contest duration in mammals (fallow deer, *Dama dama*)^17^ the results were opposite to the set hypothesis that laterality would reduce fight duration. In the current study, the negative relationship between loser lateralization strength and contest duration, is consistent with our hypothesis that strongly lateralized losers will retreat sooner. This implies that stronger lateralization resulted in better assessment of the situation. It may, however, be the case that the most dominant or strongest contestant uses its preferred side while forcing the weaker opponent to use its potentially less preferred side. Contests between pigs are characterized by opponents locking in a certain position and changing position would lead to a risk of being attacked at weaker body parts. This would imply that, at least in part, the loser’s lateralization is related to side preferences of the winner.

In line with the above, it could be argued that animals in more strongly lateralized contests terminated the contest sooner due to bite injuries (skin lesion) being accumulated more intensely on the same area of the body. Recordings of the number of skin lesions received during the contest on the left and right side of the body separately did not significantly differ from each other (data not shown), making it less likely that this is the case.

Contrary to our initial hypothesis, winners and losers did not differ in their strength or direction of lateralization. This implies that lateralization does not contribute to pigs’ RHP. This was assessed with the appropriate experimental design to test this, with opponents matched for body weight, currently the best validated proxy measure for RHP in pigs^42^. In invertebrates, asymmetries in aggressive behaviour have been related to fight success, with left biased aggression in the olive fruit fly (*Bactrocera oleae*) increasing fight success compared to right biased aggression^19^, whereas a right bias for aggression in blowfly (*Calliphora vomitoria*) resulted in increased fight success^21^. As mentioned earlier, the position of the opponents depends on each other. This may obscure the effects of individual lateralization bias. To overcome this we would recommend a paradigm in which aggressive behaviour is elicited without the physical contact of an opponent, as for example in experimental studies in fish using a mirror^43,44^.

Strongly lateralized winners tended to spend less time on display behaviour. Display behaviour is characterised mainly by parallel walking. During parallel walking one animal starts trotting in the same direction as the opponent with the head held high, to which the opponent responds by matching changes in direction by the initiator and adopting a head-up posture (Fig. 4). Lateralization during parallel walking has been shown in fallow deer (*Dama dama*), revealing a right-sided bias for this contest behaviour^15^. Parallel walking has been proposed to allow the gathering of information about the fighting ability of the opponent^17,45^. It is therefore not surprising that lateralization tended to affect display behaviour above other behaviours such as fighting. It should however be considered that the initiator is likely to start from its preferred side whereas the opponent has no choice but to respond from the opposite side which may be less preferred. Here, winner lateralization may therefore be leading, as winners are more likely to initiate agonistic behaviour^46^.

Pigs did not show population level lateralization for the combined total of observations or for specific agonistic behaviours over which a laterality index was calculated. A recent study reported that pigs do show a right-biased population level lateralization for their tail position and a majority of the pigs showed individual level lateralization when using their snout^47^. Thus, although pigs do show lateralization across several motor functions they seem to not be lateralized for agonistic behaviour at the population level. This includes that they do not show a preference for attacking when facing the opponent with the left eye, whereas in other species this preference for attacking from the left has been shown (e.g. toads, lizards, chicks, baboons, wild horses)^5^. The integration of lateralization into evolutionary biology has led to the hypothesis that population level lateralization in social species may arise as an evolutionarily stable strategy (ESS) to enable behavioural coordination^48,49^. Here the lack of evidence of population-level lateralization for agonistic behaviour is consistent with agonistic encounters favouring unpredictability and a lack of coordination between competing individuals^12^.

Males and females did not differ in their direction or strength of lateralization, with the exception that females orientated their right eye more often towards the opponent during display behaviour than males. In addition, females tended to be more strongly lateralized than males during display behaviour. In other species profound differences between males and females in strength and direction of laterality have been reported, which have been suggested to be related to the influence of corticosterone (in birds), oestrogen and testosterone^5^. The pigs in this study were pre-pubertal and it might be that sex differences in lateralization would appear predominantly at a later age.

Lateralization has increasingly been mentioned in relation to animal welfare^34,35^. Hereby it has been proposed that animals with more dominant right hemispheric use, expressed by preferences to use the left side of the body, may experience more stress given the same conditions and may respond more strongly^34^. This has been assumed through the link of the right hemisphere with the sympathetic nervous system and a bias towards more negative emotions, aggression, fear and depression. We have found no evidence for such a link in pigs but given that aggression is an important welfare issue in pig husbandry this would merit further research.

We have shown that lateralization in agonistic behaviour can contribute to the more rapid termination of a dyadic contest. Lateralization did not differ between winners and losers, suggesting that lateralization does not contribute to an individual’s fighting ability. The relationship between lateralization in agonistic behaviour and contest duration could be due to increased processing of information and deserves more research attention in the study of animal contests. We recommend future studies on aggression to capture the positioning or eye use of animals to gauge laterality, particularly as this is a non-invasive measure readily open to behavioural observation.

## Methods

### Ethical note

This study was carried out in accordance with the recommendation in the European Guidelines for accommodation and care of animals, UK Government DEFRA animal welfare codes, and adhered to the ASAB (Association for the Study of Animal Behaviour) guidelines. The work was approved by SRUC’s Animal Ethics Committee (number ED AE 21–2014) and the UK Government Home Office under the Animals Scientific Procedures Act 1986 and was conducted in constant collaboration with SRUC’s veterinary surgeon. Strict end-points were in place for the contest (described in the section ‘Contests’) but none of the thresholds were reached in the current study. Animals had no other injury than skin lesions as a result of bites, and such lesions heal within 24 h without the need for medical intervention.

### Animals and housing

A total of 104 young pigs (Large White × Landrace sow × American Hampshire boar) were studied (55 males; 49 females). Males were not castrated and the tail and teeth were kept intact. Piglets were raised in conventional farrowing crates until 4 weeks of age, after which they were weaned and relocated to the experimental building in their litter group. The experimental building had pens measuring 1.9 × 5.8 m (~1.1 m^2^/pig) with a solid floor covered with long straw (~5 kg). Pens were cleaned daily and provided with ~3.5 kg of fresh straw. Pigs had *ad libitum* access to water and pelleted commercial feed and they were weighed individually at 10 wk of age.

### Contests

Contests were staged between weight-matched pairs of pre-pubertal pigs at 10 wk of age which were unfamiliar to each other. When unfamiliar pigs meet they immediately engage in agonistic behaviour to establish a dominance hierarchy. Males and females were matched randomly, resulting in 12 female-female, 15 male-male, and 25 male-female contests. The contest arena was an indoor area which measured 2.9 × 3.8 m. The room was lit by nine overhead fluorescent strip lights providing 80–110 lux. The arena had four solid walls and had a light bedding of wood shavings covering the solid floor. To allow equal competition between the opponents, there were no objects or resources in the arena and no established territories (none had entered the arena previously). Opponents entered the contest arena simultaneously from opposite sides. The time was started from the moment both had entered the arena and timing was stopped when a clear winner was apparent, which was when one pig retreated after having received an aggressive act and failed to retaliate within 2 min after retreat. End-points for severity included a threshold of (a) injuries covering at least 75% of the body; (b) a fear response or escape attempt; (c) repeated mounting behaviour (3 times); or (d) a maximum time of 30 min. These thresholds were not reached during the observed contests.

### Video analyses

Contests were recorded in real time using a Canon Legria HF52 camera with wide angle lens mounted five meters directly above the arena. The duration of the total contest and the percentage of the contest time spent on non-contact display behaviour, non-damaging aggression, and damaging aggression were analysed by a single observer using The Observer XT 10 (Noldus, The Netherlands). Lateralization, behaviour, and specific body positions (configurations) were recorded by scan sampling both contestants every 10 seconds from the start until the end of the contest using VLC media player. For every scan sample the side with which a pig faced its opponent (left/right) was noted, including the behaviour (display behaviour, non-damaging aggression, or damaging aggression; see ethogram Table 1) and the position the opponents had towards each other. The configuration (as pictured in Fig. 1) could either be parallel (bodies lined up in the same direction with the angle between individuals <90°), head-to-head (heads together with an angle between the bodies of >90° but <270°), or head-to-tail (shoulder of one in line with the rear of the opponent). Data were obtained only when opponents were in physical contact or showed an interest in each other through opponent-directed behaviour (e.g. non-contact display). If this was not the case then the scan was considered as ‘other’ and was excluded from the analysis. If in a contest an animal was pushed against the wall, and thus forced in a certain direction, then the image was discarded. In total 1119 scan samples were obtained, with on average 18 ± 1 observations per individual (range 5–57). In addition, every unilateral bite (i.e. each bite outside a fight), the onset of the first fight within a contest, and the retreat of the loser were observed for their direction (left/right) towards the opponent. These were recorded as point events.

### Laterality index

A laterality index e.g.^50^ (LI) was calculated as ${\rm{L}}{\rm{I}}=\frac{{\rm{t}}{\rm{i}}{\rm{m}}{\rm{e}}{\rm{s}}\,{\rm{r}}{\rm{i}}{\rm{g}}{\rm{h}}{\rm{t}}\,{\textstyle \mbox{--}\mbox{--}}\,{\rm{t}}{\rm{i}}{\rm{m}}{\rm{e}}{\rm{s}}\,{\rm{l}}{\rm{e}}{\rm{f}}{\rm{t}}}{{\rm{t}}{\rm{i}}{\rm{m}}{\rm{e}}{\rm{s}}\,{\rm{r}}{\rm{i}}{\rm{g}}{\rm{h}}{\rm{t}}\,+\,{\rm{t}}{\rm{i}}{\rm{m}}{\rm{e}}{\rm{s}}\,{\rm{l}}{\rm{e}}{\rm{f}}{\rm{t}}}$. This gives a value between −1 (left eye dominant) and +1 (right eye dominant). The LI provides information on the direction to which an animal is lateralized. The strength of lateralization was evaluated by the absolute laterality index (ABLI). The ABLI is the LI standardized towards one side, converting all numbers to a positive value between 0 and +1, to indicate the strength of lateralization (0: no lateralization; 1 maximal lateralization). LI (and from that the ABLI) were also calculated for each behavioural category separately (display; non-damaging aggression; damaging aggression) as different behaviours may relate to different hemispheric activity, as well as for the left/right orientation in the parallel configuration. The minimum cut off point for calculating a LI and ABLI was considered five observations. The animal was excluded from analysis when fewer than five scan samples were available for either a contest, a behaviour or body position. This resulted in excluding 46 observations for display behaviour; 44 for damaging aggression; and 14 for the parallel configuration. Non-damaging aggression, the head-to-head position and the head-to-tail position were removed from analyses completely due to the majority of animals having fewer than 5 observations for these categories.

### Data analyses

Data were analysed with SAS Inc. 9.3 (SAS Institute Inc, Cary, USA). Data are presented as means with standard errors. Continuous data (LI and durations) were checked for normality of the residuals and were transformed if required to obtain a normal distribution. Accordingly, total contest duration was log transformed and the percentage of time spent on non-damaging aggression (duration rather than LI, which was excluded) was arcsine square root transformed. Contestants will inevitably change their position in accordance with the position of the other, and contestants’ LI are therefore not independent. To overcome this, dyad was the experimental unit except for the direct relationship between lateralization and individual sex and body weight (described below).

### Analyses at the individual animal level

Individual preferences for left or right were determined by calculating the z-score *(L−(L* + *R/2)/√((L* + *R)/4)*^14^ based on the LI for each individual. A z-score ≥ 1.96 or ≤ −1.96 was considered as right or left significantly differing (*P* < 0.05) from the sample mean. Evidence for population-level lateralization was tested through one-sample t-tests and differences in the frequency of left and right orientation were tested in paired t-tests. Differences in lateralization between winners and losers were analysed using paired t-tests. The relationship between lateralization and sex and body weight was assessed in a mixed model (PROC MIXED) with the LI of interest as the dependent variable and sex and individual body weight as fixed effects. Batch (i.e. group of animals in the trial at the same time) and litter (i.e. explaining the genetic similarity between siblings and at the same time the pen effect) were included as random effects.

### Analyses at the dyad level

Contest duration and the percentage of time spent on mutual behaviours (listed in Table 1) are by nature the same for both opponents, as well as the absolute laterality index for the parallel position. Because opponents were weight matched (<5% difference in weight) an average weight was calculated for each dyad to obtain a single variable per dyad. Male-male fights have been shown to last longer and to be more injurious than female-female and male-female fights^30^ and therefore the combination of sexes was included as a fixed effect. Contest duration and time spent on behaviours were analysed as dependent variables in a mixed model (PROC MIXED). The fixed variables were the ABLI of the winner and the ABLI of the loser, the male-female combination (FF; MF; MM) and the dyad body weight. Batch, litter (i.e. pen) of the loser, and litter of the winner were included as random effects.

Not all contests escalated into a mutual fight. To assess if lateralization affects the likelihood of a fight, fight occurrence (binary) was analysed in a Generalized Linear Mixed Model (PROC GLIMMIX) with a binary distribution and LOGIT link function. The fixed variables were the overall ABLI of the loser (ABLI calculated over the complete contest), overall ABLI of the winner, male-female combination, and dyad body weight. Batch, litter (i.e. pen) of the loser, and litter of the winner were included as random effects. Weight and sex were omitted from the models where it improved the model fit as assessed through the AIC and BIC, where models with the lowest AIC and BIC value are preferred.

## Data Availability

The datasets generated during and/or analysed during the current study are available on request from the corresponding author.
